# Tranexamic acid for patients with traumatic brain injury: a randomized, double-blinded, placebo-controlled trial

**DOI:** 10.1186/1471-227X-13-20

**Published:** 2013-11-22

**Authors:** Surakrant Yutthakasemsunt, Warawut Kittiwatanagul, Parnumas Piyavechvirat, Bandit Thinkamrop, Nakornchai Phuenpathom, Pisake Lumbiganon

**Affiliations:** 1 Surgical Unit, Khon Kaen hospital, Khon Kaen, Thailand; 2 Department of Biostatistics and Demography, Faculty of Public Health, Khon, Kaen University, Khon Kaen, Thailand; 3 Department of Surgery, Faculty of Medicine, Prince of Songkla University, Hadyai, Songkla, Thailand; 4 Department of Obstetrics and Gynaecology, Faculty of Medicine, Khon Kaen University, Khon Kaen, Thailand

**Keywords:** Traumatic brain injury, Adults, Moderately severe TBI, Intracranial haemorrhage, Progressive haemorrhage, Delayed haemorrhage, Expanding haemorrhage, Antifibrinolytic agent, Tranexamic acid, Randomized controlled trial, Human, Placebo

## Abstract

**Background:**

Traumatic brain injury (TBI) is commonly accompanied by intracranial bleeding which can worsen after hospital admission. Tranexamic acid (TXA) has been shown to reduce bleeding in elective surgery and there is evidence that short courses of TXA can reduce rebleeding in spontaneous intracranial haemorrhage. We aimed to determine the effectiveness and safety of TXA in preventing progressive intracranial haemorrhage in TBI.

**Methods:**

This is a double blinded, placebo controlled randomized trial. We enrolled 238 patients older than 16 years with moderate to severe TBI (post-resuscitation Glasgow Coma Scale (GCS) 4 to 12) who had a computerized tomography (CT) brain scan within eight hours of injury and in whom there was no immediate indication for surgery. We excluded patients if they had a coagulopathy or a serum creatinine over than 2.0 milligrams%. The treatment was a single dose of 2 grams of TXA in addition to other standard treatments. The primary outcome was progressive intracranial haemorrhage (PIH) which was defined as an intracranial haemorrhage seen on the second CT scan that was not seen on the first CT scan, or an intracranial haemorrhage seen on the first scan that had expanded by 25% or more on any dimension (height, length, or width) on the second scan.

**Results:**

Progressive intracranial haemorrhage was present in 21 (18%) of 120 patients allocated to TXA and in 32 (27%) of 118 patients allocated to placebo. The difference was not statistically significant [RR = 0.65 (95% CI 0.40 to 1.05)]. There were no significant difference in the risk of death from all causes in patients allocated to TXA compared with placebo [RR = 0.69 (95% CI 0.35 to 1.39)] and the risk of unfavourable outcome on the Glasgow Outcome Scale [RR = 0.76 (95% CI 0.46 to 1.27)]. There was no evidence of increased risk of thromboembolic events in those patients allocated to TXA.

**Conclusions:**

TXA may reduce PIH in patients with TBI; however, the difference was not statistically significant in this trial. Large clinical trials are needed to confirm and to assess the effect of TXA on death or disability after TBI.

## Background

Each year, world-wide more than 1.5 million people die and about 10 million people are hospitalized following traumatic brain injury (TBI) [1]. Many survivors experienced long term disability [2]. In Thailand, each year around 8,000 people died following acute TBI and tens of thousands were hospitalized. A follow up study of 418 patients who were admitted to Khon Kaen regional hospital following TBI found that 29% of patients were either dead or severely disabled six months following the injury [3].

TBI is commonly accompanied by intracranial bleeding which occurs in 25% to 45%, 3% to 12% and 0.2% of severe, moderate and mild TBI cases respectively [4]. There were evidences indicating that this bleeding can develop or continue after hospital admission and that larger bleeds have a worse prognosis [5]–[12]. Delayed enlargement of traumatic intraparenchymal contusions and hematomas is the most common cause of clinical deterioration and death in patients who had a lucid interval after TBI [8,13].

A systematic review of randomized controlled trials (RCT) shows that antifibrinolytic agents are effective in reducing bleeding in elective surgery. The review summarized data from 211 RCTs involving 20,781 participants showed that both Aprotinin and Tranexamic acid (TXA) reduced the need for blood transfusion in elective surgery by about one third [RR = 0.61 (95% CI 0.54 to 0.69)] with no evidences of increased adverse effects [14]. If antifibrinolytics reduced intracranial bleeding in patients with TBI, this would have important clinical implications. However, a systematic review of haemostatic drugs in patients with TBI found no RCTs [15].

There were some evidences on the use of TXA in patients with spontaneous aneurysmal intracranial bleeding. A systematic review of RCTs of TXA in aneurysmal haemorrhage found that although rebleeding was reduced by 45% [odds ratio = 0.55 (95% CI 0.42 to 0.71)], this benefit was offset by cerebral ischaemia such that there was no overall patients benefit [16]. However, these trials used high doses TXA for about six weeks and it has been suggested that a shorter and lower dose might prevent rebleeding whilst avoiding the risk of ischaemia. Indeed, since the review was published a trial of early short course (3 days) TXA showed that it reduced rebleeding from 10.8% to 2.4% without ischaemic adverse effects [17].

We conducted a double blind randomized controlled trials to evaluate the effect of early short course TXA on the occurrence of progressive intracranial haemorrhage (PIH) in patients with TBI treated at a large regional trauma centre in rural Thailand.

## Methods

A randomized, double-blind, placebo-controlled, parallel group trial was conducted. The trial protocol was approved by the Khon Kaen hospital’s ethics committee and the Khon Kaen University’s ethics committee. The trial protocol was registered with http://www.clinicaltrials.gov with the trial identifier NCT00755209. All written informed consent forms were obtained from legally acceptable representative of comatose participants.

### Participants

All patients, older than 16 years, with moderate to severe TBI (post-resuscitation Glasgow Coma Scale (GCS) 4 to 12) who had a computerized tomography (CT) brain scan performed within eight hours of injury, and whom there was no immediate indication for surgery, were eligible for inclusion. There were both of isolated TBI and polytrauma patients whom there were critical concern for TBI management during the admission period. Patients were excluded if they were pregnant, had evidences of coagulopathy, known to be receiving a medication which affects haemostasis, or had a serum creatinine over than 2 mg/decilitre. Coagulopathy was considered present if any of the following hematological parameters were observed: (1) platelet count less than 100,000 cells/mm3; (2) Prothrombin time (PT) or international normalized ratio (INR) prolonged more than 1.5 times normal value; (3) activated partial thromboplastin time (aPTT) more than 10 seconds greater than normal value. Coagulopathy was a risk factor for developing PIH and mortality in previous studies [10,11,18]–[22] or recent reports [23,24]. Hence it was confounding factor to be controlled by exclusion.

### Intervention

Patients were randomly allocated to receive TXA (loading dose of 1.0 gram over 30 minutes followed by a maintenance dose of 1.0 gram infused over eight hours) or matching placebo. The placebo was sterile water and was purchased on the open market in Thailand.

### Study outcomes

The primary outcome was progressive intracranial haemorrhage. It would have more association to therapeutic effect of given tranexamic acid than other outcomes in this study [25]. Moreover it was a significant source or link to morbidity and mortality in TBI [8,10,18]–[20,26]. PIH was defined as an intracranial haemorrhage seen on the second CT scan that was not seen on the first CT scan, or an intracranial haemorrhage seen on the first scan that had expanded by 25% or more on any dimension (height, length, or width) on the second scan. Progressive pressure effect was defined as either an increase in midline shift of greater than 1 mm or an increase in basal cistern between the first and second CT scan. The second CT scan was to be taken 24 hours ± 8 hours after the first CT scan. Improved Glasgow Coma Scale (GCS) motor score was also recorded to see if there is compatible change between clinical and radiological progression at 24 hours.

The presence or absence of PIH was assessed by two independent readers. Both were neurosurgeons at KKH with experiences in reading posttraumatic CT scans. When there was disagreement about the presence or absence of PIH this was resolved by a third neurosurgeon reader. Inter-rater reliability was assessed by kappa statistic.

Secondary outcomes were death, functional status assessed using the Glasgow Outcome Scale (GOS) at hospital discharge, blood transfusion, neurosurgical operation and any in-hospital thromboembolic events (myocardial infarction, pulmonary embolism, deep vein thrombosis, and stroke).

### Sample size

We planned to randomize approximately 240 patients, 120 to each group. We estimated that the proportion of patients with PIH would be 30% in the placebo group and that TXA would reduce to be 15%. A trial with 240 patients would have about 80% power at the 0.05 level of significance (two-sided test) to detect a treatment effect of this magnitude.

### Randomisation and blinding

The randomisation sequence (with a randomly varied block size) was generated from a computer by a person who was not involved with the trial and this sequence was used to prepare the sequentially numbered treatment packs. Whenever an eligible patient was recruited, the recruiting clinician asked that the next sequentially numbered sealed opaque treatment pack be opened and that the trial loading dose and maintenance infusion be prepared and sent to the relevant ward. This preparation was done out of sight of the recruiting clinician and research participants by nurses who were not involved in the trial. Each treatment pack contained unlabelled vials of either drug or placebo. Although drug and placebo vials contained an identical amount of colorless solution, there was a small size discrepancy between the drug and placebo vials. It was for this reason that the vials were enclosed within sequentially numbered sealed opaque envelopes that were opened by nurses who were not involved in the trial. This approach ensured good allocation concealment and also ensured that those caring for the patient and those conducting the trial did not know the assigned treatment. The allocation scheme was kept confidential from all research participants until the end of the study.

### Statistical methods

The primary analysis was on an intention-to-treat (ITT) basis with complete case analysis for other outcomes and was done using STATA software version 10.0. The presence or absence of PIH was analyzed as a dichotomous variable. The Glasgow Outcome Scale was also dichotomized such that death, persistent vegetative state, and severe disability constituted an unfavorable outcome while favorable outcome included moderate disability and good recovery. We calculated relative risks, risk difference with their 95% confidence intervals and hypothesis testing between the two treatment groups.

## Results

### Patient recruitment

**Figure 1:**
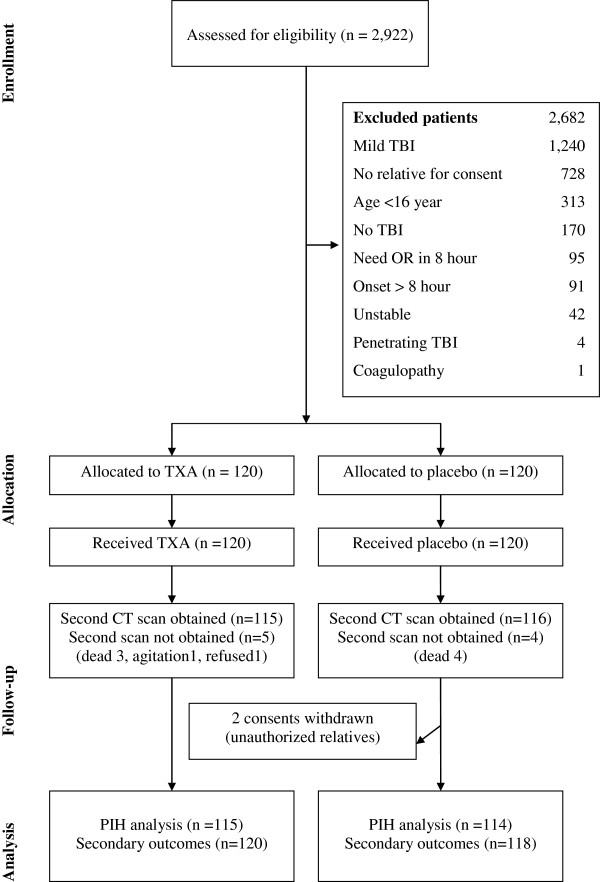
Participant flow.

Figure 1 shows the participant flow into the trial. The first patient was recruited on the 23rd October 2008 and the last patient on 14th August 2009 by which time a total of 238 patients had been included in the trial. All patients received the allocated trial treatments (TXA or placebo) and there were no protocol violations. There were nine patients for whom a second CT scan could not be obtained: seven patients died before the second CT scan, one patient could not be scanned because of agitation, and one patient refused the second scan. There were two consents withdrawal in the placebo group after randomization because they were signed by the unauthorized relatives. The related ethic committees were informed with an agreement for this exclusion. The inter-rater reliability of the assessment of the presence or absence of PIH was high with a kappa statistic of 0.95. The patients were enrolled with comparable profile including about mean age (40 years), male gender (80%), injury onset (within 7 hours), associated organ injury with injury severity score 24 (range from 9–43) and initial haematocrit level (38 volumes%) with moderately severe GCS severity. There were similar pressures effects finding of the first CT scan in both groups. Treatment and control groups were approximately balanced with respect to baseline characteristics (Table 1).

**Table 1:** Baseline characteristics

**Characteristics**	**TXA (n =120)**	**Placebo (n = 118)**
Age at randomization (years)	34.8 (16.0)	34.1 (15.3)
Male	103 (86%)	107 (91%)
Glasgow coma scale (GCS) severity: Moderate (9 – 12) Severe (4 – 8)	52 (53%)	47 (47%)
	68 (49%)	71 (51%)
Baseline haematocrit (volume %)	38 (7.4)	38 (6.7)
Time since injury (hours)	6.6 (1.69)	7.1 1.29)
Isolated TBI injury	20 (17%)	16 (14%)
Polytrauma with TBI injury	100 (83%)	102 (86%)
Mean injury severity score (range)	23.3 (9–43)	24.8 (9–43)
Midline shift (>3 mm) on first CT (mm)	2 (0.02%)	3 (0.03%)
Basal cistern compression on first CT	54 (45%)	53 (45%)

Table 2 shows the effect of TXA on the study outcomes by the intention to treat analysis with assuming poor outcome which is used to represent the effectiveness of treatment effect. Progressive intracranial haemorrhage was present in 21 (18%) of patients allocated to TXA and in 32 (27%) of patients allocated to placebo. The difference was not statistically significant [RR = 0.65 (95% CI 0.40 to 1.05)]. The relative risk of death from all causes in patients allocated to TXA compared with placebo was 0.69 (95% CI 0.35 to 1.39) and the relative risk for unfavourable outcome on the Glasgow Outcome Scale was 0.76 (95% CI 0.46 to 1.27). The relative risk of blood transfusion need in patients allocated to TXA compared with placebo was 0.92 (95% CI 0.61 to 1.40). Although we had informed the clinical condition and proposed for emergency neurosurgical operation in all patients with PIH to their relatives. The neurosurgical interventions were not done in placebo group because the patients’ relatives did not allow us to perform the operation hence the relative risk for neurosurgical interventions could not be calculated. There were very few adverse effects in both groups. There was no patient in TXA group who developed vascular occlusion event in this study.

**Table 2:** Study outcomes

**Outcomes**	**TXA**	**Placebo**	**RD**	**RR**
	**(n = 120)**	**(n = 118)**	**[95% CI]**	**[95% CI]**
Progressive intracranial haemorrhage (PIH)	21 (18%)	32 (27%)	−0.10	0.65
moderate TBI (n = 24)	7 (6%)	17 (14%)	[(−0.20)-	[0.40-1.05]
severe TBI (n = 20)	9 (8%)	11 (9%)	(−0.01)]	
indicated neurosurgery	6 (5%)	6 (5%)		
Increase in pressure effect*	*11 (10%)	12 (11%)	−0.01	0.91
			[(−0.09) – 0.07]	[0.42-1.97]
Improved GCS motor score at 24 hours	37 (31%)	37 (31%)	−0.01	0.98
			[(−0.12) – 0.11]	[0.67-1.44]
Neurosurgical intervention	3 (3%)	0	0.03	---
			[(−0.00) – 0.05]	---
Blood products transfusion	31 (26%)	33 (28%)	−0.02	0.92
			[(−0.13) – 0.09]	[0.61-1.40]
Death	12 (10%)	17 (14%)	−0.04	0.69
			[(−0.13) – 0.04]	[0.35-1.39]
Unfavorable (GOS) outcome	21 (18%)	27 (23%)	−0.05	0.76
			[(−0.16) – 0.05]	[0.46-1.27]
**Adverse events**				
Stroke	0	3	---	---
Pulmonary embolus	0	0	---	---
Deep vein thrombosis	0	0	---	---
Gastrointestinal bleeding	0	1	---	---

*Denominator for outcomes is 114 for TXA group and 115 for placebo group. This is an analysis based on complete case analysis that is not assumed the missing outcome.

Table 3 shows the effect of various methods of handling missing response in the primary outcome. The different analyses have different assumptions on existing PIH. A complete case analysis may be undertaken for primary outcome to avoid the assumption about PIH without imputation for the unavailable outcome. However different analyses give similar trend of potential benefit for treatment with tranexamic acid.

**Table 3:** Effect of various methods of handling missing response in trial

**Methods of analysis**	**PIH in TXA and placebo group**				
	**Group**	**n/N**	**Rate**	**RD**	**RR**
		**(person)**	**(%)**	**[95% CI]**	**[95% CI]**
1. Complete case analysis	TXA	16/115	14%	−0.11	0.57
	Placebo	28/114	25%	[(−0.21)-(−0.01)]	[0.32-0.99]
2. Assuming poor outcome	TXA	21/120	18%	−0.10	0.65
	Placebo	32/118	27%	[(−0.20)-(−0.01)]	[0.40-1.05]
3. Assuming good outcome	TXA	16/120	13%	−0.10	0.56
	Placebo	28/118	24%	[(−0.20)-(−0.01)]	[0.32-0.98]
4. Extreme case favoring placebo	TXA	21/120	18%	−0.06	0.74
	Placebo	28/118	24%	[(−0.16)-0.04]	[0.44-1.22]
5. Extreme case favoring TXA	TXA	16/120	13%	−0.14	0.49
	Placebo	32/118	27%	[(−0.24)-(−0.04)]	[0.29-0.85]

## Discussion

### Principal findings

We found a statistically insignificant difference but with a trend towards significant reduction in the risk of PIH in patients allocated to TXA without evidence of increased risk of thrombotic adverse events. The risks of death and of unfavorable outcome on the Glasgow Outcome Score were lower for patients allocated to TXA. The safety of early short course treatment of TXA in our TBI patients was compatible to no increasing risk of non-fatal vascular occlusive events with early short course treatment of TXA in traumatic bleeding patients in CRASH2 trial [27].

### Strengths and weaknesses

This is the leading randomized trial to examine the effectiveness of the early administration of a short course of TXA in patients with acute TBI. We are aware of a recent study publication of CRASH2 trial and we anticipate that the results of all relevant trials will ultimately be combined in the Cochrane systematic review of haemostatic drugs for TBI [15]. Our trial was properly randomized with good allocation concealment. The timing of the second CT scan was pre-specified in the protocol and all outcome measurements were made without knowledge of treatment allocation. The main weakness of our study is the low power to estimate the effect of TXA on clinical outcomes. In particular, although our study has shown statistically insignificant difference that TXA may reduce PIH, because of the low powers to examine the effect of TXA on death and on disability, the clinical implications of our findings are still limited.

Another limitation is lacking to explore an effect of associated injury or injury severity score in multiple trauma patients which was concerned in some studies [19,21]. We precluded exploring this association to the treatment by our limited sample size. Polytrauma subjects were almost patients whom there was agreement from trauma care team to concern treatment for TBI as same as isolated TBI subjects at first moment. Therefore we assigned serious, critical and serious injury degree for moderate TBI, severe TBI and other anatomical organ injuries in the category of injury severity score assessment respectively. The common associated injuries were extremity fracture, facial fracture, rib or clavicular fracture and a few cases of other chest injury or minor abdominal trauma.

Finally the exclusion of coagulopathy is also a limitation of our study and this subject could be a point to explore in further research.

### PIH in TBI

Various terms have been used to describe the development or enlargement of intracranial bleeding after TBI. Terms include delayed traumatic intracranial haemorrhage (DTICH) [7], expanding hematoma [19] and progressive hemorrhagic injury [10]. In this paper we combined these concepts such that we included both new haemorrhage and expanding haemorrhage (progressive intracranial haemorrhage). Our rationale was that both lesions may exacerbate intracranial hypertension and the occurrence of both may be affected by the administration of TXA.

Although patients were excluded if they had evidence of coagulopathy at baseline the proportion of patients with PIH among the patients was surprisingly high. Had we not excluded coagulopathic patients it is likely that the proportion of patients with PIH would have been even higher. Previous estimates of the occurrence of PIH vary widely from 7% to 60% [20]. These differences are likely to reflect difference in timing of the CT scan, clinical setting and diagnostic criteria. Nevertheless, given the relatively high prevalence of PIH in patients with TBI observed in this study the potential for TXA to improve clinical outcomes could be high.

### Methods of handling missing response in trial

We summarize the result of handling missing outcome for the primary outcome in Table 3.

## Conclusions

We have not shown that TXA improves clinical outcomes and this information would be required in order to make any recommendation about the use of TXA in clinical practice. Our results have important implications for research. If an early short course of TXA could be demonstrated to improve clinical outcomes after TBI without any important adverse effects, then this treatment, because it is cheap and widely practicable, could contribute importantly to reducing mortality and disability after TBI.

## Competing interests

The authors declare that they have no competing interests.

## Authors’ contributions

SY, BT, NP and PL designed, analysed and wrote the paper. WK and PP suggested the design, collected the data and conducted analyses. SY wrote the paper with input from all other authors. All authors had full access to all the data in the study and had final responsibility for the decision to submit for publication. All authors read and approved the final manuscript.

## Pre-publication history

The pre-publication history for this paper can be accessed here:

http://www.biomedcentral.com/1471-227X/13/20/prepub
